# Voltage-Dependent Anion Channel-1, a Possible Ligand of Plasminogen Kringle 5

**DOI:** 10.1371/journal.pone.0164834

**Published:** 2016-10-17

**Authors:** Yin-ku Liang, Liu-jiao Bian

**Affiliations:** 1 College of Life Sciences, Northwest University, Xi’an 710069, P. R. China; 2 College of Biological Science and Engineering, Shaanxi University of Technology, Hanzhong 723000, P. R. China; 3 Shaanxi Province Key Laboratory of Bio-Resource, Shaanxi University of Technology, Hanzhong 723000, P. R. China; 4 Qinba Mountains of Bio-Resource Collaborative Innovation Center of Southern Shaanxi province, Shaanxi University of Technology, Hanzhong 723000, P. R. China; University of Akron, UNITED STATES

## Abstract

Kringle 5, the fifth fragment of plasminogen, is known to be important for inhibiting the proliferation and migration of vascular endothelial cell (VEC), while not having any effects on normal endothelial cells. Therefore, it may be a potential tumor therapy candidate. However, the ligand of the Kringle 5 in VEC has not yet been identified. In this study, the possible ligand of Kringle 5 in vitro was screened and validated using Ph.D.-7 phage display peptide library with molecular docking, along with surface plasma resonance (SPR). After four rounds of panning, the specific clones of Kringle 5 were confirmed using enzyme-linked immunosorbent assay (ELISA). The gene sequence analysis showed that they expressed the common amino sequence IGNSNTL. Then, using a NCBI BLAST, 103 matching sequences were found. Following the molecular docking evaluation and considering the acting function and pathway of the plasminogen Kringle 5 in the human body, the most promising candidate was determined to be voltage-dependent anion channel-1 (VDAC-1), which was able to bind to Kringle 5 at -822.65 J·mol^-1^ of the binding energy at the residues of Lys12, Thr19, Ser57, Thr188, Arg139, Asn214, Ser240 and Lys274. A strong dose-dependent interaction occurred between the VDAC-1 and Kringle 5 (binding constant 2.43 × 10^3^ L·mol^-1^) in SPR observation. Therefore, this study proposed that VDAC-1 was a potential ligand of plasminogen Kringle 5, and also demonstrated that the screening and validation of protein ligand using phage display peptide library with the molecular docking, along with SPR, was a practicable application.

## Introduction

The previous investigations on tumors have chiefly focused on gene structures and function variations. The treatment strategies have been to kill the tumor cells directly. Recently, increased attentions have been paid to the influence of tumor microenvironments on the formation, differentiation, evolution, metastasis, and invasion of a tumor, such as a tumor vessel and an extracellular matrix. In fact, studies show that a tumor microenvironment may be a crucial target for cancer treatment, as well as preventative strategies. Endostatin has been emerging as an intriguing chemotherapeutic target and Kringle 5 has been found to be an effective endostatin-based drug. Kringle 5, which is the fifth kringle domain of plasminogen is highly similar to other kringle domains and is composed of approximately 80 amino acid residues [1^,^ 2]. Kringle 5 has high specificity in inhibiting the proliferation of VEC and its inhibition ability is significantly greater than that of endostatin found early [3, 4, 5]. Kringle 5 mainly inhibits VEC proliferation by causing a stagnation of the VEC growth cycle, which in turn induces apoptosis. However, Kringle 5 does not have any effect on other cells, such as fibroblast, human stem cells, and so on [5, 6]. Kringle 5 can also inhibit the hyperplasia of blood vessel by down-regulating the vascular endothelial growth factor (VGEF), and up-regulating the pigment epithelium-derived factor (PEDF) [7]. Studies have certified that kringle 5 effectively inhibit the growth and stimulate the apoptosis of VEC by binding with them. Nevertheless, the target of the kringle 5 in VEC has not yet been identified. Therefore, this study mainly focuses on the binding protein with Kringle 5.

The methods for screening the ligand of protein, such as cDNA Library technology, yeast-two-hybrid technology and immune-precipitation, [8, 9, 10] have been used to study antibodies, oligonucleotides, ligands and receptors. Phage display peptide library can be used to screen antibodies and small molecular ligands, due to its simplicity and low cost. However, it has not been used to screen macromolecular ligand and protein receptors. Therefore, the technology of screening the ligand of protein using phage display peptide library in combination with molecular docking and SPR was reported in this study. These data lay a foundation for understanding the potential of screening receptor or ligand of protein using phage display peptide library and molecular docking technology.

## Materials and Methods

### Materials and Reagents

Strains and recombinant plasmids pCMV6-XL5-VDAC-1 containing hVDAC-1 cDNA (GenBank accession No.BC039893) fragment were purchased from OriGene Technologies. The prokaryotic expression vector pET-28b (Germany, No.3713185), *Escherichia coli* DH5α competent cells (China, No.MCC001), BL21 (DE3) (Germany, No.1660) competent cells, and M13 phage extraction kit were purchased from the Beijing Dingguochangsheng Biotechnology Co., Ltd. The Ph.D.-7 phage display peptide library kit (UK, No.E8102L) was purchased from New England Bio-labs, Inc. The pET-28b-hK5 expression vector was built in our laboratory. The Ni^2+^-chelated Sepharose 6 Fast Flow (10 × 58 mm) and Sephadex G25 pre-packed column (10 × 100 mm) were purchased from GE Healthcare Life Sciences (Uppsala, Sweden).

The agarose (SPAIN, No.111855) and tryptone (SPAIN, No.L0042B-500) were purchased from the Gene Company Ltd. The isopropyl b-D-1-thiogalactopyranoside (IPTG) (Germany, No.420322) was purchased from Merck & Co., Inc. X-Gal (USA, No.X1000-05) was purchased from US Biological. The N, N-dimethylformamide (CAS:68-12-2) was purchased from the Jining Hengtai Chemical Industry Co., Ltd. Golden View (China, No.EP1501) was purchased from the Bioteke Co., Ltd. The DL2000 DNA Marker (China, No.GD1305-250UL) and Middle DNA Marker I (China, No.NMW006) were obtained from the Beijing Dingguochangsheng Biotechnology Co., Ltd. The premix PrimeSTAR DNA polymerase (Japan, No.R050B), *Nhe*I endonucleases (Japan, No.1160BH), *Xho*I endonucleases (Japan, No.1010AH), and T4 DNA ligase (Japan, No.2011A) were purchased from the Takara Co., Ltd. The plasmid mini kit (China, No.12123) and gel extraction kit (China, No.28704) were purchased from the Omega Co., Ltd. The low molecular weight protein marker (USA, No. 26610) was purchased from the Thermo Co., Ltd. The ampicillin salt (USA, No.0219452605) and kanamycin (USA, No.K001-10) were purchased from MPBIO. The other main reagents were domestic pure analysis reagents.

### Methods

#### Expression and purification of plasminogen Kringle 5

The recombinant plasmid pET-28b-hK5 was constructed and transformed into *E*. *coli* BL21 (DE3) competent cells. A mono-clone which was selected from the transformed plates was inoculated in 5 mL LB culture medium containing 50 μg·mL^-1^ kanamycin. The mixture was cultured in a shaker at 37°C 220 rpm for 12 hours. The following day, 1 mL of the culture medium was inoculated in 100 mL of fresh LB culture medium containing 50 μg·mL^-1^ kanamycin. The mixture was incubated at 37°C and 220 rpm until the OD_600_ was 0.4–0.6. After adding 0.8 mmol·L^-1^ IPTG, the mixture was further cultured at 37°C and 220 rpm for 8 hours. Then, the bacteria were harvested by centrifugation, and mixed with an ultrasonic crushing lysis buffer (pH 8.0, 10.0 mmol·L^-1^ Tris-HCL, 5.0 mmol·L^-1^ EDTA-Na_2_) at a ratio of 1 g: 10 mL. The precipitate was removed by centrifugation at 6,000 rpm for 30 minutes at 4°C following ultrasonication. The supernatant was applied to a Ni^2+^-chelated Sepharose 6 Fast Flow column which had been equilibrated with a dialysis buffer A (10.0 mmol·L^-1^ Tris-HCl, pH 8.0, containing 5.0 mmol·L^-1^ EDTA-Na_2_). Then, the column was eluted with 90% buffer A and 10% buffer B (buffer A, plus 500 mmol·L^-1^ imidazole, pH8.0), and the fraction of interest was collected by eluting the column using 50% buffer A and 50% buffer B. Finally, the fractions of interest were collected and desalted with a Sephadex G-25 pre-packed column (10×100 mm), and their molecular weights were analyzed by SDS-PAGE. The dimension of the Ni^2+^-chelated Sepharose 6 Fast Flow column was 10×58 (mm), the flow-rate of elution solution was 1.0 mol·L^-1^ and the UV adsorption was detected at 280 nm.

#### Screening Ph.D.-7 phage display peptide library

An ELISA plate was coated with 100 μL 150 μg·mL^-1^ Kringle 5 diluted with a sodium bicarbonate buffer (0.1 mol·L^-1^ NaHCO_3_, pH 8.6), incubated overnight at 4°C. In the following day, the coating solution was removed from ELISA plate. Then the ELISA plate was filled with blocking buffer (0.1 μg·mL^-1^ streptavidin, 5 mg·mL^-1^ BSA, 0.2 mg·mL^-1^ NaN_3_), and incubated for at least two hours at 4°C. Additionally, one uncoated well should be blocked as the control group. The blocking solution was discarded, and the plate was washed ten times with TBST (TBS + 0.1% [v/v] Tween-20). At this point, a 10-fold representation of the library diluted with TBS (0.15 mmoL·L^-1^ NaCl, pH 7.5 50 mmol·L^-1^ Tris-HCL) buffer was pipetted into the coated plate. The plate was rocked gently at room temperature for 30 minutes. The nonbinding phage was discarded by pouring off and slapping the plate face-down onto a clean paper towel, and the plate was washed ten times with TBST (TBS + 0.1% [v/v] Tween-20). The phage which was able to bind to the Kringle 5 was eluted from the plate with 100 μL 0.2 M Gly-HCL (pH 2.2, 0.1% BSA). The eluate was pipetted into a micro-centrifuge tube, and neutralized with 15 μL of 1 mol·L^-1^ Tris-HCL (pH 9.1). 1 μL was assayed and the rest of it was amplified. The screening processes were repeated four times, including gradually raising the concentration of Tween-20 during the wash steps, gradually reducing the coating concentration of Kringle 5, and gradually shortening the incubation times during the coating steps, from the second to fourth rounds of the screening.

#### ELISA

One row of ELISA plate wells for each clone were coated with 100 μL of 150 μg·mL^-1^ Kringle 5 and incubated overnight at 4°C in an air-tight humidified box. Next day, the excess Kringle 5 solution was removed from the plate, and the plate was blocked with blocking buffer (0.1 μg·mL^-1^ streptavidin, 5 mg·mL^-1^ BSA, 0.2 mg·mL^-1^NaN_3_) at 4°C for one hour. Additionally, one row of uncoated wells per clone to be characterized should be blocked as the control group. After removing the blocking buffer and washing ten times with TBST (TBS + 0.1% [v/v] Tween-20), 100 μL of serial dilution phage clone was added to ELISA plate, and incubated at room temperature for two hours. The excess phage clone was removed, and the plate was washed ten times with TBST. Then, the wells of the plate were filled with 200 μL anti-M13 antibodies (diluted to 1:5000 in the sealing solution) which was marked with horse radish peroxidase (HRP), and shaken at room temperature for one hour. After removing the anti-M13 antibodies, the plate was washed ten times with TBST, and 200 μL of a 3, 3′, 5, 5′-tetramethylbenzidine (TMB) substrate solution was added to each well. The entire system was kept at room temperature for 15 minutes, until the color changed. Finally, 50 μL of 2 moL·L^-1^ H_2_SO_4_ was used to terminate the reaction, and the absorbance at 410 nm was measured using a microplate reader.

#### Sequencing of phage DNA

*E*.*coli* ER2738, which had been infected with the positive phages, was cultured on a LB agar plate coated with IPTG/X-gal. The individual plaques were randomly selected and amplified, and the phages DNA were isolated and sequenced by Sangon Biotech (Shanghai, China).

#### Homology analysis and molecular docking

After the peptide sequence was conformed, the sequence of the selected peptide was compared with the anthropogenic protein database using the NCBI Basic Local Alignment Search Tool (BLAST) and the homologous proteins of the selected peptide were obtained. Then, the crystal structures of the homologous proteins were found from the NCBI, or predicted using Modeller 9.10 software. The Modeller 9.10 software was also used to predict the crystal structure of the Kringle 5, with 5HPG as a template [11]. A molecular docking with a HEX software program was used for a flexible docking process.

#### Expression and purification of VDAC-1

The primers were designed according to the VDAC-1 cDNA sequence (NM_003374) and the ends of the VDAC-1 cDNA sequence were inserted into the restriction enzyme sites of *Xho*I and *Nhe*I, respectively. The recombinant plasmid pCMV6-XL5-VDAC-1 was the template. The upstream and downstream primers (F, R) and the high fidelity Premix DNA polymerase were used to amplify the VDAC-1 cDNA sequence. The PCR procedures were as follows: 98°C pre-denaturation for 5 minutes; 98°C denaturation for 30 seconds, 60°C for 30 seconds, 72°C for 30 seconds for 30 cycles, and a final 72°C extension for 10 minutes. The PCR products were analyzed by 1.5% agarose gel electrophoresis, retrieved and purified using a gel extraction kit. The retrieved target fragments and plasmid pET-28b were double digested with the *Xho*I and *Nhe*I restriction enzymes, respectively. The digested fragments and plasmids were retrieved with a gel extraction kit, and incubated overnight with T4 ligase at a constant temperature of 16°C. The ligation products were transformed into competent cells of *E*. *coli* DH5α. The transformed DH5α were cultured overnight on a LB solid medium containing 50 μg·mL^-1^ kanamycin. In the following day, the positive clones were cultured, and the plasmids were extracted and identified by the double enzyme digestions. The enzyme digestion conditions were as described above. The positive clones were sent for sequencing to the Shanghai Biological Engineering Co., Ltd. (China).

The recombinant plasmid which was verified correctly by the sequencing was named pET-28b-hVDAC-1. Then, it was transformed into *E*. *coli* BL21 (DE3) competent cells for the expression of the recombinant proteins. A mono-clone was selected from the transformed plates, and inoculated in 5 mL LB liquid medium containing 50 μg·mL^-1^ kanamycin. Then the recombinant bacteria were cultured in a shaker at 37°C at 220 rpm for 12 hours. The following day, 1 mL of culture was inoculated in 100 mL fresh LB medium containing 50 μg·mL^-1^ kanamycin. The mixture was cultured at 37°C and 220 rpm until OD_600_ = 0.5. After adding 1.0 mmol·L^-1^ IPTG, the mixture was cultured at 35°C at 220 rpm for 8 hours. The induced and cultured *E*. *coli* fermentation liquid was centrifuged for harvesting the bacterial precipitate, and was mixed with an ultrasonic crushing lysis buffer (pH 8.0, 10.0 mmol·L^-1^ Tris-HCL, 5.0 mmol·L^-1^ EDTA-Na_2_) at a ratio of 1 g: 10 mL. Following the ultrasonic processing, the precipitate was collected. A denaturant solution buffer (6 mol·L^-1^ guanidine hydrochloride, 10 mmol·L^-1^ β-mercaptoethanol, 5 mmol·L^-1^ EDTA-Na_2_, 10 mmol·L^-1^ Tris-HCl, pH 8.0) was added to the precipitate at a ratio of 1 g: 5 mL. The supernatant was collected after centrifugation at 10,000 rpm for 30 minutes. The renatured solution buffer (10 mmol·L^-1^ Tris-HCL, 5 mmol·L^-1^ EDTA-Na_2_, 1 mmol·L^-1^ reduced glutathione, 0.2 mmol·L^-1^ oxidized glutathione, pH 8.0) was added into the denaturing solution at a ratio of 20: 1 (V/V) for 24 hours. The refolded samples were centrifuged, and a protein supernatant sample containing renatured recombinant hVDAC-1 was obtained. Finally, the renatured recombinant hVDAC-1 was loaded and purified on the Ni^2+^-chelated Sepharose 6 Fast Flow column. The elution buffer A (20 mmol·L^-1^ phosphate buffer, pH 8.0) was first used to equilibrate the Ni^2+^-chelated affinity column. Then a gradient elution programmer for the elution buffer B (0.50 mol·L^-1^ imidazole, 20 mmol·L^-1^ phosphate buffer, pH 8.0) from 0 to 100% within 150 minutes was used to isolate the protein supernatant sample containing recombinant hVDAC-1. The dimension of the Ni^2+^-chelated Sepharose 6 Fast Flow column was 10×58 (mm), the flow-rate of elution solution was 1.0 mol·L^-1^ and the UV adsorption was detected at 280 nm.

#### SPR

The Kringle 5 was coupled to a CM5 sensor chip using an amino coupling reagent kit. The cells which did not couple were served as a reference surface, in order to correct for the systemic noise and minor responses of the nonspecific binding. The CM5 sensor chip was activated with a 7-minutes injection of 0.05 mol·L^-1^ NHS and 0.2 mol·L^-1^ EDC, followed by a 7-minutes injection of VDAC-1 dissolved in 10 mmol·L^-1^ PBS (5% DMSO, pH 4.5). All of the coupling procedures were performed at 25°C, and a flow rate of 40 μl·min^-1^. The regeneration of the surfaces was achieved by an injection for 120 seconds of 10 mmol·L^-1^ HEPES, pH 7.4, and 300 mmol·L^-1^ NaCl. Each of the VDAC-1 samples was injected over the surface in duplicate, and each experiment was repeated three times on two separately produced immobilization surfaces.

#### Statistical analysis

Statistical analyses were performed using version (SAS INSTITUTE INCIBM). All experimental results were obtained from three independent experiments and were presented as the mean ± standard deviation (SD). Student′s *t*-test was applied to analysis experimental results and P <0.05 was considered significant.

## Results

### Expression and purification of plasminogen Kringle 5

**Fig 1:**
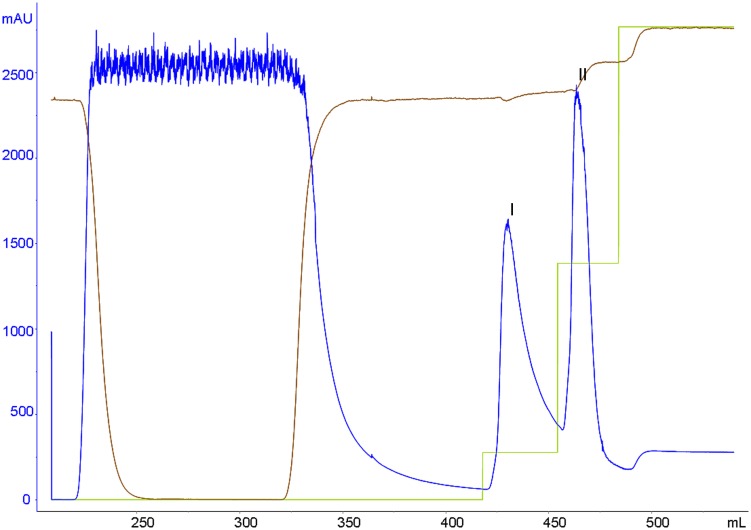
Chromatogram of separating plasminogen Kringle 5 supernatant solution. The supernatant was purified with Ni^2+^-chelated Sepharose 6 Fast Flow column which had been equilibrated with a dialysis buffer A (10.0 mmol·L^-1^ Tris-HCl, pH 8.0, containing 5.0 mmol·L^-1^ EDTA-Na_2_). PeakI was eluted with 90% buffer A and 10% buffer B (buffer A, containing 500 mmol·L^-1^ imidazole, pH8.0), and PeakII was eluted with 50% buffer A and 50% buffer B.

**Fig 2:**
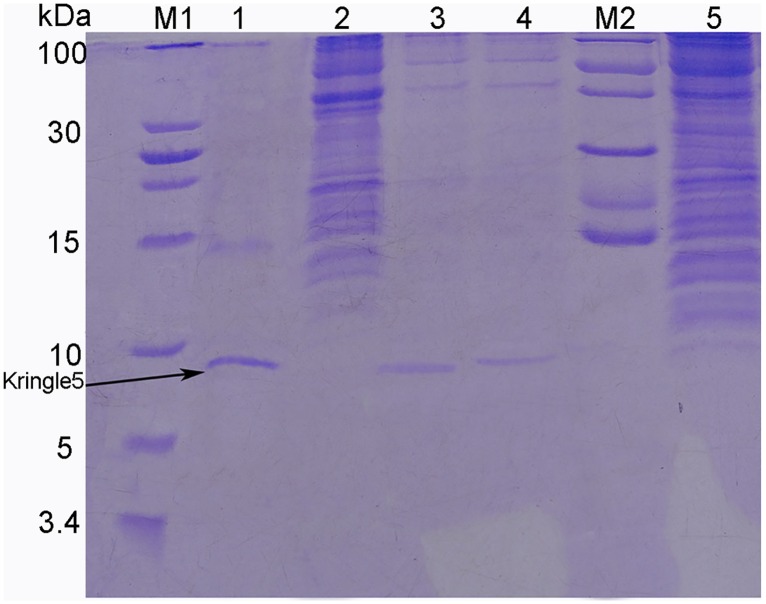
Tris-tricine SDS-PAGE analysis of the collecting part. (M1): Protein Low-MW marker (from top to bottom MW were 100, 30, 25, 20, 15, 10, 5 and 3.4 kD). (M2): Protein MW marker (from top to bottom MW were 116, 66.2, 45, 35, 25, 18 and 14.4 kD). (1): Collection of PeakII (Fig 1) was purified with sephadex G-25 column and then analyzed by Tris-tricine-SDS-PAGE electrophoresis. (2): Protein eluted with the mixture solution of 90%A and 10%B (PeakI, Fig 1). (3, 4): Protein eluted with the mixture solution of 50%A and 50%B (PeakII, Fig 1). (5): Flow-through proteins not binding to the Ni^2+^-chelated Sepharose 6 Fast Flow column.

Induction and expression experiments showed that recombinant plasminogen Kringle 5 containing a 6 × his tag sequence expressed in *E*. *coli* BL21 (DE3) was soluble. The plasminogen Kringle 5 supernatant was separated and purified on a Ni^2+^-chelated Sepharose 6 Fast Flow column. Two elution peaks could be identified through successive elution with buffers A and B, respectively (Fig 1). The SDS-PAGE showed that there was no the band of the marker with a molecular weight of 8 kDa in elution peak I (Fig 2). But there was the band of the marker with a molecular weight of 8 kDa in elution peaks II (Fig 2). Therefore, it was inferred that the recombinant plasminogen Kringle 5 was mainly included in the elution peak II. Determining its purity and concentration, the results showed that the purity of the obtained recombinant plasminogen Kringle 5 was approximately 91% according to the densitometry quantification using BandScan software, and the concentration was (190.89 ± 4.48) μg·mL^-1^ by BSA as a standard.

### Screening plasminogen Kringle 5 affinity peptides

Through a high binding ELISA plate coated with recombinant plasminogen Kringle 5, we could screen the affinity ligand of plasminogen Kringle 5 by panning Ph.D.-7 phage display peptide library (Table 1). Our preliminary experiments found that changing the conditions of panning could improve the screening efficiency. Therefore, four rounds of panning were implemented, with a gradual lowering of the concentration of Kringle 5 coated to the well of the plate, a gradual shortening of the incubation times, and a gradual increasing of the concentration of tween-20, which offered a yield coefficient of 6.25 × 10^−9^. The yield coefficient was increased from 3.33 × 10^−8^ in the second screening round to 1.57 × 10^−7^ in the fourth screening round, which indicated that the pool of phage became enriched in favor of the sequences that bind to the Kringle 5(Table 1).

**Table 1:** Recovery rate of phages from four rounds of screening.

Rounds	Input phage (pfu)	Output phage (pfu)	Recovery (%)
1	2.0×10^10^±2.35×10^8^	1.25×10^2^±35	6.25×10^−9^
2	2.3×10^11^±3.30×10^9^	7.93×10^3^±78	3.33×10^−8^*
3	4.3×10^11^±7.50×10^9^	1.18×10^4^±1.25×10^3^	2.73×10^−8^*
4	8.1×10^10^±2.79×10^9^	1.29×10^4^±7.87×10^2^	1.57×10^−7^*

*: T-test was applied to analysis experimental results and P <0.05 was regarded as statistically significant for all tests.

The phage recovery was obtained using the ratio of input and output concentrate.

### DNA sequencing and affinity analyzing of positive phages

After four rounds of panning, fifty well-separated individual phage clones were then randomly selected from the sample group LB culture plates, on which the total number of phage clones were no more than 100. As shown in Table 2, the sequencing data revealed that 18 phage clones had the same amino acid sequence (IGNSNTL), accounting for about 40% of the total number of samples; 4 phage clones had the same amino acid sequence (LKLGEKW); 4 phage clones had the same amino acid sequence (LPWQIHN); 4 phage clones had the same amino acid sequence (CGNSNTL); 2 phage clones had the same amino acid sequence (NTGSPYE); 2 phage clones had the same amino acid sequence (NMQITKG) and other phage clones express different short peptides. They were all named Ph.D.-7-1 to Ph.D.-7-15, respectively (Table 2). Therefore, the amino acid sequence which occurred in the highest frequency was IGNSNTL.

**Table 2:** Gene and amino acid sequences of monoclonal phages screened by Kringle 5.

Series	Sample Numbers	Gene sequence	Amino acid sequence	Names
1	2,3,4,5,6,8,10,11,13,15,17,21,23,25,29,39,40,49	GTC TCA TAA TCT TAA TGG TTA	IGNSNTL	Ph.D.-7-1
2	9,12,28,44	GGT GAA GAG TGG TTC GAA TTC	LKLGEKW	Ph.D.-7-2
3	20,36,45,48	TAA TAC TTA GAC GGT TCC GTT	LPWQIHN	Ph.D.-7-3
4	14,30,38,47	GTC TCA TAA TCT TAA TGG TTA	CGNSNTL	Ph.D.-7-4
5	22,24	GAG TAT TCC GCT TGG TCA TAA	NTGSPYE	Ph.D.-7-5
6	31,35	TGG GAA GCA TTA GAC GTA TAA	NMQITKG	Ph.D.-7-6
7	1	GGT GAA GAC GAA GTG GCA TCC	PTVLQLT	Ph.D.-7-7
8	7	GCT TGC TCA TAC GAG TCA TGA	STEHTRS	Ph.D.-7-8
9	16	GTT TAC TAC GAC TAA GCA GCC	PTNQHHL	Ph.D.-7-9
10	18	TCA TAT GAC TCA TGA GAA GTG	VKSTQYT	Ph.D.-7-10
11	19	GAA TTT TCG GGG TTG TCC GTA	MPVGAFK	Ph.D.-7-11
12	26	TGC GCG GAA TAC GTT GAG TAG	DELHKAR	Ph.D.-7-12
13	42	GCT GAC TTG GCA TGG TTC GAG	ELGTVQS	Ph.D.-7-13
14	46	TAC TCC GCA TTT GCG GGG TTT	FGAFTPH	Ph.D.-7-14
15	50	GAA TCT GCA GTT GAG TAC TTT	FHELTSK	Ph.D.-7-15

Fifty well-separated blue plaques were randomly selected from the LB culture plates of sample group and sequenced. The sequencing data revealed that samples (27 32 33 34 37 41 43) were empty vector and did not express any 7 peptide.

At the same time, twenty well-separated individual phage clones were randomly selected from the control group LB plates, on which the total number of phage clones were no more than 100. The sequencing data showed that 20 phage clones expressed four different short peptides, Ph.D.-7-C-1 to Ph.D.-7-C-4, as shown in Table 3. Comparing the amino acid sequences of sample group (Table 2) with the amino acid sequences of control group (Table 3), we found that Ph.D.-7-C-1 and Ph.D.-7-14 had the same amino acid sequence, Ph.D.-7-C-2 and Ph.D.-7-11 had the same amino acid sequence, and Ph.D.-7-C-4 and Ph.D.-7-9 were also the same. Therefore, Ph.D.-7-9, Ph.D.-7-11 and Ph.D.-7-14 were determined to be false positives and should been eliminated from the sample group.

**Table 3:** Gene and amino acid sequences of monoclonal phages of control group.

Series	Control Number	Gene sequence	Amino acid sequence	Names
1	1,8,9,11	TAC TCC GCA TTT GCG GGG TTT	FGAFTPH	Ph.D.-7-C-1
2	2,10,12,13,16,17,18	GAA TTT TCC GGG TTG TCC GTA	MPVGAFK	Ph.D.-7-C-2
3	3,5,6,7	TCA GAG GGG GTT TCT TTC GAA	KLSLGET	Ph.D.-7-C-3
4	4,14,15,19,20	GTT TAC TAC GAC TAA GCA GCC	PTNQHHL	Ph.D.-7-C-4

**Fig 3:**
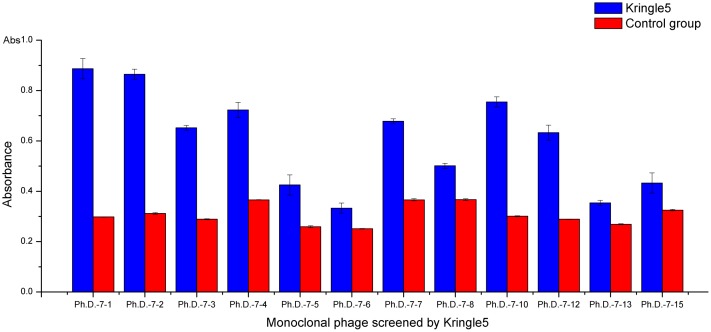
Affinity analysis of plasminogen Kringle 5 to screening phages. One row of ELISA plate wells coated with Kringle 5 per clone was sample group which were indicated by blue. One row of ELISA plate wells coated with nothing per clone was control group were indicated by red. The absorbance of sample group and control group at 410 nm was measured using a microplate reader.

The affinity of these short peptides (excluding false positives) for Kringle 5 was tested using ELISA. The sample was larger twice higher than that of control group. The results indicated that six positive phage clones (Ph.D.-7-1, Ph.D.-7-2, Ph.D.-7-3, Ph.D.-7-4, Ph.D.-7-10, Ph.D.-7-12) had a high affinity for Kringle 5(Fig 3). Furthermore, Ph.D.-7-1 and Ph.D.-7-4 had the same amino acid sequence (GNSNTL). Therefore, the GNSNTL sequence was believed to be the likely binding site for the Kringle 5. Comparing the affinity of Ph.-D.-7-1 for Kringle 5 with the affinity of Ph.-D.-7-4 for Kringle 5, we found that the affinity of Ph.-D.-7-1 for Kringle 5 was higher than that of Ph.-D.-7-4. So the IGNSNTL might have been the binding site for Kringle 5.

### Sequence alignment

By comparing the seven peptides (IGNSNTL) in the NCBI anthropogenic protein database, 103 proteins were revealed. These proteins were divided into twenty-three types according to the structure and function of them. Considering the acting function of these proteins in the human body, we found that there were eleven kinds of proteins which were more likely to be the ligands of plasminogen Kringle 5 (Table 4). Furthermore, through considering and assessing the acting function and pathway of the plasminogen Kringle 5 in the human body, we speculated that the most likely ligand of plasminogen Kringle 5 was voltage-dependent anion channel-1 (VDAC-1), a beta-barrel mitochondrial outer membrane protein and played an important role in the regulation of mitochondrial functions and the control of apoptosis.

**Table 4:** Eleven proteins containing binding sequences specific for Kringle 5 and the binding energy for Kringle 5 with them.

Seven peptides	Protein	Accession number	E value	Max ident	Binding energy(J·mol^-1^)
IGNSNTL	Laminin subunit alpha-3 isoform 2 precursor	NP_000218.3	131	100%	-566.83
	ATP-binding cassette sub-family	NP_775099.2	131	100%	-726.34
	Nipped-B-like protein	XP_005248339.2	131	100%	-563.25
	MDS1 and EVI1 complex locus protein	NP_005232.2	132	100%	-425.8
	Protein C-ets-2	NP_001243224.1	132	100%	-268.23
	Piezo-type mechanosensitive ion channel component 2	NP_071351.2	188	83%	-458.79
	Mitogen-activated protein kinase kinase kinase 13 isoform 1	NP_004712.1	188	100%	-563.21
	Cochlin precursor	NP_004077.1	188	100%	-389.12
	Voltage-dependent anion-selective channel protein 1	NP_003365.1	189	100%	-822.65
	Transmembrane channel-like protein 2	NP_542789.2	383	83%	-524.72
	Endothelin B receptor isoform X2	XP_005266332.2	550	86%	-337.53

### Molecular docking

**Fig 4:**
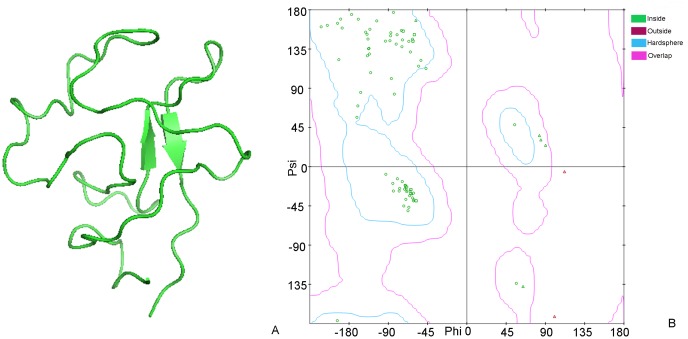
3D images (A) and the Ramachandran plot (B) of the Kringle 5. The crystal structure of the Kringle 5 was constructed with 5 HPG as a template by the Modeller 9.10.

**Fig 5:**
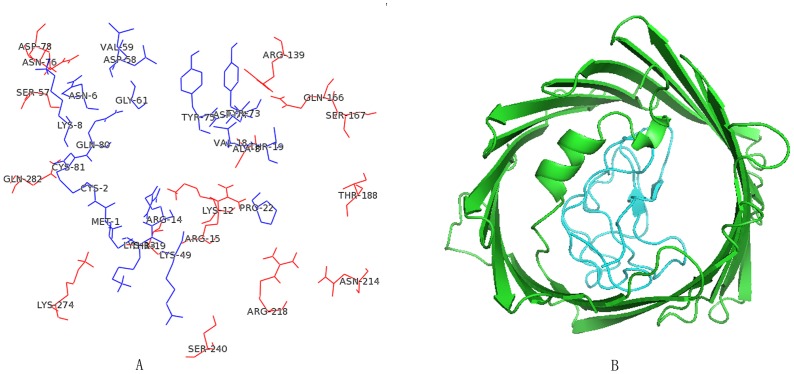
2D (A) and 3D (B) images of the binding complex between plasminogen Kringle 5 and VDAC-1. The amino acid residuals and tertiary structure of VDAC-1 bound to plasminogen Kringle 5 were indicated by red (A) and green (B), respectively. And the amino acid residuals and tertiary structure of plasminogen Kringle 5 bound to VDAC-1 were indicated by blue (A) and wathet blue (B), respectively.

**Fig 6:**
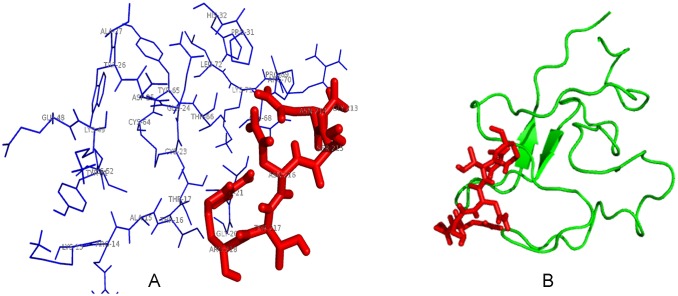
2D (A) and 3D (B) images of the binding complex between plasminogen Kringle 5 and GNSNT of VDAC-1. The amino acid residuals and tertiary structure of GNSNT of VDAC-1 bound to plasminogen Kringle 5 were indicated by red (A, B). And these of plasminogen Kringle 5 bound to the GNSNT of VDAC-1 were indicated by blue (A) and yellow green (B), respectively.

Molecular docking is a new technology to investigate the protein-protein interaction, and we can find interaction protein by predicting the stability of combination between protein and protein. According to this theory, we studied the stability of combination between these eleven proteins and Kringle 5 using molecular docking. We constructed the crystal structures of Kringle 5 and these eleven proteins using Modeller 9.10 or obtained the crystal structures of protein from the NCBI. The crystal structure of the Kringle 5 was constructed with 5 HPG which had been constructed as the full amino acids sequence of Kringle 5 in 1998 as a template by the Modeller 9.10. It was composed of two β-pleated sheets and a random coil [11] (Fig 4A). The Ramachandran plots showed that seventy five amino acids (92.59%) were present in a “allowed region”, four amino acids (4.90%) were present in a “maximum allowed area”, only two amino acids (2.50%) were represent in a “generously allowed region” (Fig 4B). These results suggested that the final structure of Kringle 5 was reasonable. The interactions between Kringle 5 and these eleven proteins were analyzed using HEX software. A lower binding energy was used to select the interacting proteins with Kringle 5, and the binding energy for VDAC-1 with Kringle 5 was found to be -822.65 j·mol^-1^ (Table 4). The two- and three-dimensional images for the binding complex between plasminogen Kringle 5 and VDAC-1 were illustrated in Fig 5A and 5B. From Fig 5B, it was known that the two β-pleated sheets of Kringle 5 folded almost parallel occupied the entire channel of the VDAC-1, and bound with the VDAC-1 via a hydrophobic effect. The key amino acids of the VDAC-1 which bound with Kringle 5 were Lys (12), Thr (19), Ser (57), Thr (188), Arg (139), Asn (214), Ser (240) and Lys (274) (Fig 5A), and Thr, Ser and Asn appeared in the 7 peptide sequence (IGNSNTL). In analyzing the binding sites of GNSNT with VDAC-1, we found that the GNSNT could bind with Kringle 5 at the residues of Pro(31), Ala(27), Asp(25), Trp(26) and Thr(21) (Fig 6A and 6B). These results indicated that the VDAC-1 might be a ligand of the Kringle 5.

### Expression and purification of VDAC-1

**Fig 7:**
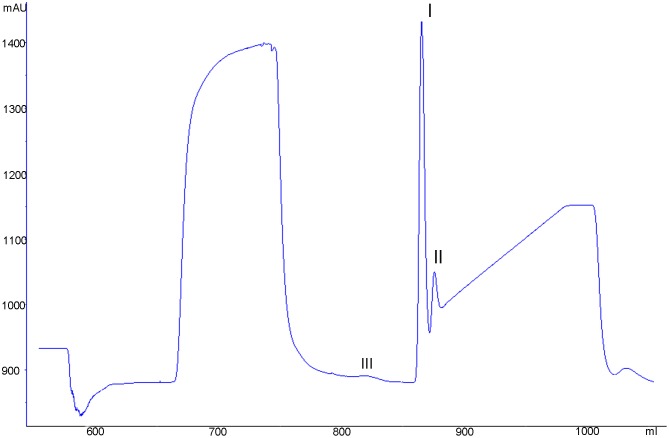
Chromatogram of separating renatured recombinant hVDAC-1. The renatured recombinant hVDAC-1 was applied to Ni^2+^-chelated Sepharose 6 Fast Flow column which had been equilibrated with a elution buffer A(20 mmol·L^-1^ phosphate buffer,pH 8.0). Then a gradient elution programmer for the elution buffer B(0.50 mol·L^-1^ imidazole,20 mmol·L^-1^ phosphate buffer, pH 8.0) from 0 to 100% within 150 min was used to elute the protein ample containing recombinant hVDAC-1. The dimension of the Ni^2+^-chelated Sepharose 6 Fast Flow column was 10×58 (mm), the flow-rate of elution solution was 1.0 mol·L^-1^ and the UV adsorption was detected at 280 nm.

**Fig 8:**
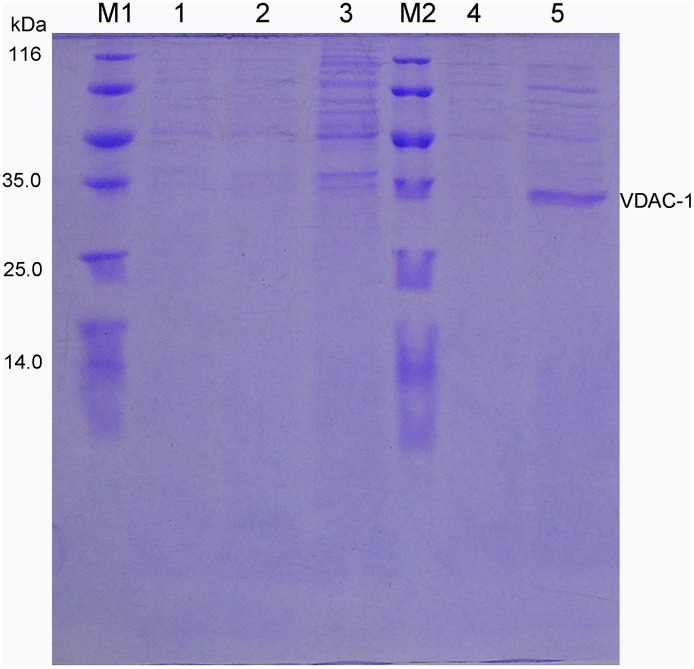
SDS-PAGE analysis of the collecting part. (M1, M2): Protein MW markers (from top to bottom the MW were 116, 66.2, 45, 35, 25, 18 and 14.4 kD); (1, 2, 4): Peak III in Fig 7 (3): Peak I in Fig 7; (5): PeakII in Fig 7.

Through induction and expression experiments, it was known that recombinant VDAC-1 containing a 6 × his tag sequence was expressed in *E*. *coli* BL21 (DE3) in an inclusion body form. The renatured VDAC-1 supernatant was isolated and purified on the Ni^2+^-chelated Sepharose 6 Fast Flow column and their elution components were analyzed by SDS-PAGE. From Figs 7 and 8, it could be found that there was no band of the marker with a molecular weight of 32 kDa in elution peak I. Therefore, no recombinant VDAC-1 was included in the elution peak I. Whereas there were the bands of the marker with a molecular weight of 32 kDa in elution peaks II, indicating that the recombinant VDAC-1 was mainly included in the elution peak II. Collecting the elution peak II and scanning its purity, we found that the purity of the obtained recombinant VDAC-1 was approximately 89% according to the densitometry quantification using BandScan software.

### Affinity of Kringle 5 and VDAC-1 by SPR-based

**Fig 9:**
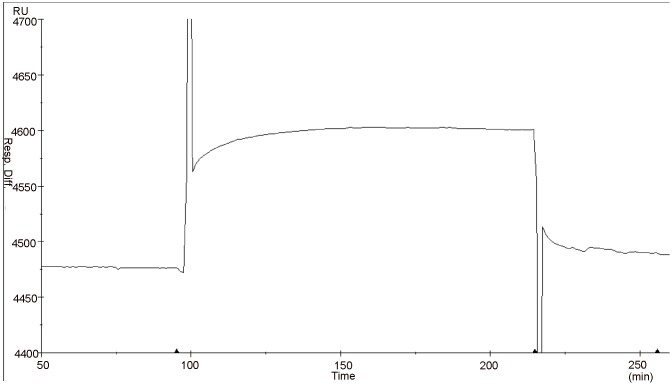
Surface plasmon resonance sensorgram of immobilized plasminogen Kringle 5.

**Fig 10:**
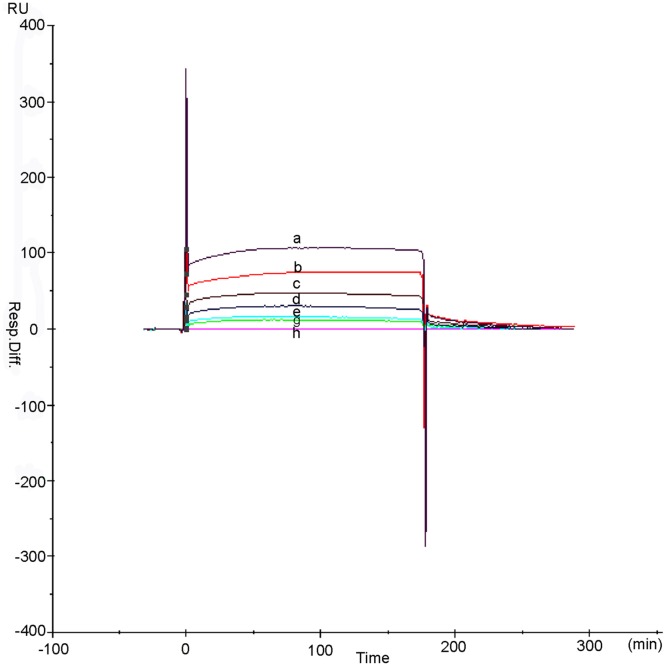
Surface plasmon resonance sensorgram of interacting between immobilized plasminogen Kringle 5 and VDAC-1. The data were representative of seven independent experiments; a, b, c, d, e, f, g and h were representative of different concentration of VDAC-1 (a: 280.00μmol·L^-1^, b: 140.00 μmol·L^-1^, c: 70.00 μmol·L^-1^, d: 35.00 μmol·L^-1^, e: 17.5 μmol·L^-1^, f: 8.25 μmol·L^-1^, g: 4.13 μmol·L^-1^ and h: 0.00 μmol·L^-1^).

The SPR-based biosensors enabled the monitoring of the interaction between the biomolecules in real time, with a label-free assay format. In order to validating the affinity of plasminogen Kringle 5 to VDAC-1, a plasminogen Kringle 5-immobilized sensor chip was used for SPR. Kringle 5 was covalently coupled to CM5 with an amino coupling reagent. The immobilization curves (Fig 9) showed that the greatest immobilization was achieved at 180 RU and the amount of VDAC-1 bound to the surface of plasminogen Kringle 5-immobilized sensor chip increased in a concentration-dependent manner (Fig 10). When the concentrations of VDAC-1 increased from 0 to 280.00 μmol·L^-1^, the amounts of the VDAC-1 bound to the sensor chip increased gradually from 0 to 110.0 RU. From the relationship between the concentrations of VDAC-1 and the amounts of VDAC-1 bound to the sensor chip, it was known that the binding constant Ka and dissociation constant Kd for the binding interaction of plasminogen Kringle 5 to VDAC-1 were 2.43×10^3^ (L·mol^-1^) and 4.12×10^−4^ (L·mol^-1^), respectively.

## Discussion

In recent years, study results from the research focusing on the understanding of the mechanism of angiogenic inhibition have been reported, and the complexity and uncertainty of this inhibition has stalled advances in the field. Currently, it has been hypothesized that angiostatin may be related to matrix metalloproteinase [12, 13], anti-apoptotic proteins Bcl-2 or Bcl-XL [14, 15], integrin α5β1 and caveolin-1[16, 17, 18, 19, 20], ATP synthase [21, 22], and endothelial growth factor receptor [23]. Previous studies on the angiogenic inhibition of Kringle 5 are related to the glucose-regulated protein78 (GRP78) on the surface of the endothelial and tumor cells [24]. GRP78 forms a complex with Procaspase-7 in cellulose tumor cells. Caspase-7 is produced by the complex degradation with Kringle 5 and induces apoptosis [25, 26]. Recently, Bui Nguyen et al. reported that Kringle 5 was able to induce endothelial cell apoptosis by activating caspase-7 [27]. Also, Mario et al. reported that Tyr252-Lys283 sites of streptokinase which interacted with human plasminogen had a high homology with voltage-dependent anion channel-1 (VDAC-1), and it was found that Kringle 5 could induce a decrease in pH and mitochondrial membrane hyperpolarization by interfering with ion channel effect [28]. Therefore, the VDAC-1 of endothelial cells may potentially be a ligand of Kringle 5. Although some of parameters of angiogenic inhibition have been established, the ligand of Kringle 5 and the simplest and effective ways to find the ligand of proteins have not been fully elucidated. Thus, for the first time, the Ph.D.-7 phage display peptide library with molecular docking, along with SPR was employed in this study to screen for protein with high affinity for Kringle 5. In addition, VDAC-1 was chosen as the ligand of Kringle 5 and VDAC-1 could interact with Kringle 5.

Through preliminary experiments, we found that it was difficult to pan peptides binding specifically to Kringle 5 using long phage display peptide library. However, after four rounds of biopanning, the Kringle 5-bound phages were significantly enriched using Ph.D.-7 phage display peptide library, which indicated that successfully biopanning were related to the active centre of protein and that long peptide library was not beneficial for mimicking the active centre of protein. So the Ph.D.-7 phage display peptide library was suitable to be used to screen the peptides binding specifically to protein. However, after sequencing, we obtained about twelve short peptides which were not good for NCBI blast searching. So high-frequency appeared and high affinity was the leading indicators for selecting the phage clone. By using this method, the short peptide (IGNSNTL) was obtained. The results of searching NCBI anthropogenic protein database using the NCBI-BLAST server revealed 103 different proteins which might be the ligands of Kringle 5. Such results were unbeneficial to subsequent validation on the interacting of these proteins with Kringle 5. And further, through considering and assessing the acting function and pathway of these proteins and the plasminogen Kringle 5 in the human body, we could speculate that there were about eleven proteins which were likely the ligands of plasminogen Kringle 5, and VDAC-1 was more likely the ligand of plasminogen Kringle 5. However, confirming the ligand of Kringle 5 only through considering and assessing the acting function and pathway of the plasminogen Kringle 5 in the human body was too hasty. So we studied the key amino acids and binding energy of the interacting protein using the molecular docking. By analyzing the key amino acids and binding energy of the interacting of Kringle 5 with these eleven proteins, the potential ligand (VDAC-1) of Kringle 5 was eventually obtained. In this process we found that the short peptide obtained using Ph.D.-7 phage display peptide library could accurately mimic the active centre of VDAC-1. But as we know, the amino acid sequence of the active centre of protein are discontinuous in most cases, which was unbeneficial for searching the NCBI protein database using this short peptide. However, if the short peptide had a high homology with a protein, the protein might be rich in these amino acids which composed the short peptide, and the active centre of the protein might be built with it, too. So comparing the amino acids of these proteins binding to Kringle 5 with the amino acid of the short peptide was very important to confirm whether it was the ligand of Kringle 5 using molecular docking. After comparing, we found the key amino acids residues of the VDAC-1 which bound to Kringle 5 were Lys (12), Thr (19), Ser (57), Thr (188), Arg (139), Asn (214), Ser (240) and Lys (274). Thr, Ser and Asn appeared in the 7 peptide sequence (IGNSNTL), accounting for about 63% of the key amino acids of VDAC-1 binding to Kringle 5 and accounting for about 57% amino acids of IGNSNTL. So we thought IGNSNTL mimicked the active centre of VDAC-1 and VDAC-1 might be the ligand of Kringle 5. SPR showed that VDAC-1 could bind to Kringle 5 (Kd, 4.12 × 10^−4^, Ka, 2.43 × 10^3^). These further evidenced that VDAC-1 might be the ligand of Kringle 5. The data of this study was consistent with previous finding [28].

## Conclusion

Screening and validating protein ligand using a random phage-display library with molecular docking, along with SPR, is a powerful and novel method for screening the ligand of target protein. Comparing with the cDNA library technology, yeast-two-hybrid technology and so on, the current method is proven to be easier and cheaper in conducting the experiments. Furthermore, it is determined that the VDAC-1 is the ligand of Kringle 5.
